# Use of Pediatric Injectable Medicines Guidelines and Associated Medication Administration Errors: A Human Reliability Analysis

**DOI:** 10.1177/1060028021999647

**Published:** 2021-03-01

**Authors:** Matthew D. Jones, Jonathan Clarke, Calandra Feather, Bryony Dean Franklin, Ruchi Sinha, Ian Maconochie, Ara Darzi, Nicholas Appelbaum

**Affiliations:** 1 University of Bath, Somerset, UK; 2 Imperial College London, UK; 3 University College London, UK; 4 Imperial College Healthcare NHS Trust, London, UK

**Keywords:** administration, intravenous, guidelines as topic, medication errors, nurses, patient safety, practice guidelines as topic

## Abstract

**Background:**

In a recent human reliability analysis (HRA) of simulated pediatric resuscitations, ineffective retrieval of preparation and administration instructions from online injectable medicines guidelines was a key factor contributing to medication administration errors (MAEs).

**Objective:**

The aim of the present study was to use a specific HRA to understand where intravenous medicines guidelines are vulnerable to misinterpretation, focusing on deviations from expected practice (*discrepancies*) that contributed to large-magnitude and/or clinically significant MAEs.

**Methods:**

Video recordings from the original study were reanalyzed to identify discrepancies in the steps required to find and extract information from the NHS Injectable Medicines Guide (IMG) website. These data were combined with MAE data from the same original study.

**Results:**

In total, 44 discrepancies during use of the IMG were observed across 180 medication administrations. Of these discrepancies, 21 (48%) were associated with an MAE, 16 of which (36% of 44 discrepancies) made a major contribution to that error. There were more discrepancies (31 in total, 70%) during the steps required to access the correct drug webpage than there were in the steps required to read this information (13 in total, 30%). Discrepancies when using injectable medicines guidelines made a major contribution to 6 (27%) of 22 clinically significant and 4 (15%) of 27 large-magnitude MAEs.

**Conclusion and Relevance:**

Discrepancies during the use of an online injectable medicines guideline were often associated with subsequent MAEs, including those with potentially significant consequences. This highlights the need to test the usability of guidelines before clinical use.

## Introduction

Medication errors are a leading cause of avoidable patient harm and cost an estimated $42 billion per annum worldwide.^1^ The administration of intravenous medicines is associated with a higher frequency of medication administration errors (MAEs) than medicines given by any other route. Up to 48% of all injectable doses may be erroneous in some way.^2,3^ Each year in the United States, there are an estimated 1.2 million hospitalizations affected by an injectable medicine error, increasing costs by $2.7 to $5.1 billion.^4^ One of many causes of such errors is difficulty finding relevant, unambiguous information in guidelines.^5-7^ Little is known, however, about the specific challenges that occur during the process of accessing and reading a guideline or why health professionals encounter such difficulties. This knowledge would be invaluable for designing safer guidelines.

A recent study examined medication errors during simulated pediatric resuscitations, using human reliability analysis (HRA) to describe how these errors were linked to discrepancies in individual process steps.^8^ As is usual in the United Kingdom, intravenous medicines were prepared at the simulated patient’s bedside. Ineffective retrieval of preparation and administration instructions from the NHS Injectable Medicines Guide (IMG) was the step that most often made a major contribution to medication errors. This is a website that provides specific guidance on the preparation and administration of more than 350 intravenous medicines and is accessed approximately 3 million times per annum.^9^ Three other studies have identified the IMG as potentially difficult to use.^10-12^ However, this previous research cannot be used to recommend improvements to guideline design because it did not analyze the precise steps in the process of accessing and reading the guidelines that were linked to medication errors.

The aim of the present study, therefore, was to reanalyze video recordings from the previous HRA study^8^ to identify the steps in the process of using the IMG that contributed to medication errors. HRA undertakes analysis of system vulnerabilities at a task level^13,14^ and was also adopted for the present study but with a detailed focus on the process of accessing and reading the IMG, rather than the entire resuscitation. The incidence, nature, and severity of the medication errors were identified in the previous study, so our specific objective here was to use HRA to understand the contributory role played by discrepancies in the guideline use process, with a focus on those discrepancies contributing to large-magnitude and/or clinically significant errors. Although the previous study considered both prescribing and administration errors,^8^ because the present study focuses on the use of medicines administration guidelines, we considered only MAEs.

## Methods

### Previous Study

The previous pediatric resuscitation simulation study was a prospective, observational study conducted in a medical simulation facility within a large academic hospital.^8^ Resuscitation teams consisting of a senior pediatric doctor (registrar or above), a junior doctor, a senior pediatric nurse (UK salary band 6 or above), and a junior pediatric nurse were randomized to complete 1 of 2 standardized scenarios: prolonged status epilepticus in an 8-month old (8 teams) or presumed meningococcal sepsis in a 10-month old (7 teams). During these 15 simulations completed by 15 different teams, 180 intravenous medicines were prepared and administered to a mannequin by the nurses. Participants had access to printed information sources, hospital information technology systems, and the IMG website.^9^ The simulations were recorded by 7 high-definition video cameras, including head-mounted cameras worn by both nurses.

The main outcome measures of the previous study were medication errors and discrepancies. Medication errors were defined as an overall error with respect to a particular medication’s administration as a whole, after having been administered to the patient. Greater than 25% deviation from either the recommended dosing range or rate of administration was considered a “large magnitude medication error.” The potential severity of every error was assessed using the Dean and Barber tool.^2,3,15^ Errors with a mean severity score >3 were considered “clinically significant errors.” Expected practice was defined using a hierarchical task analysis (HTA), and a discrepancy was defined as an observed deviation from this expectation.

The previous study was performed in line with the principles of the Declaration of Helsinki. Approval was granted by the hospital concerned and the United Kingdom’s Health Research Authority. Participants gave written, informed consent before taking part.

### Hierarchical Task Analysis

**Figure 1.**
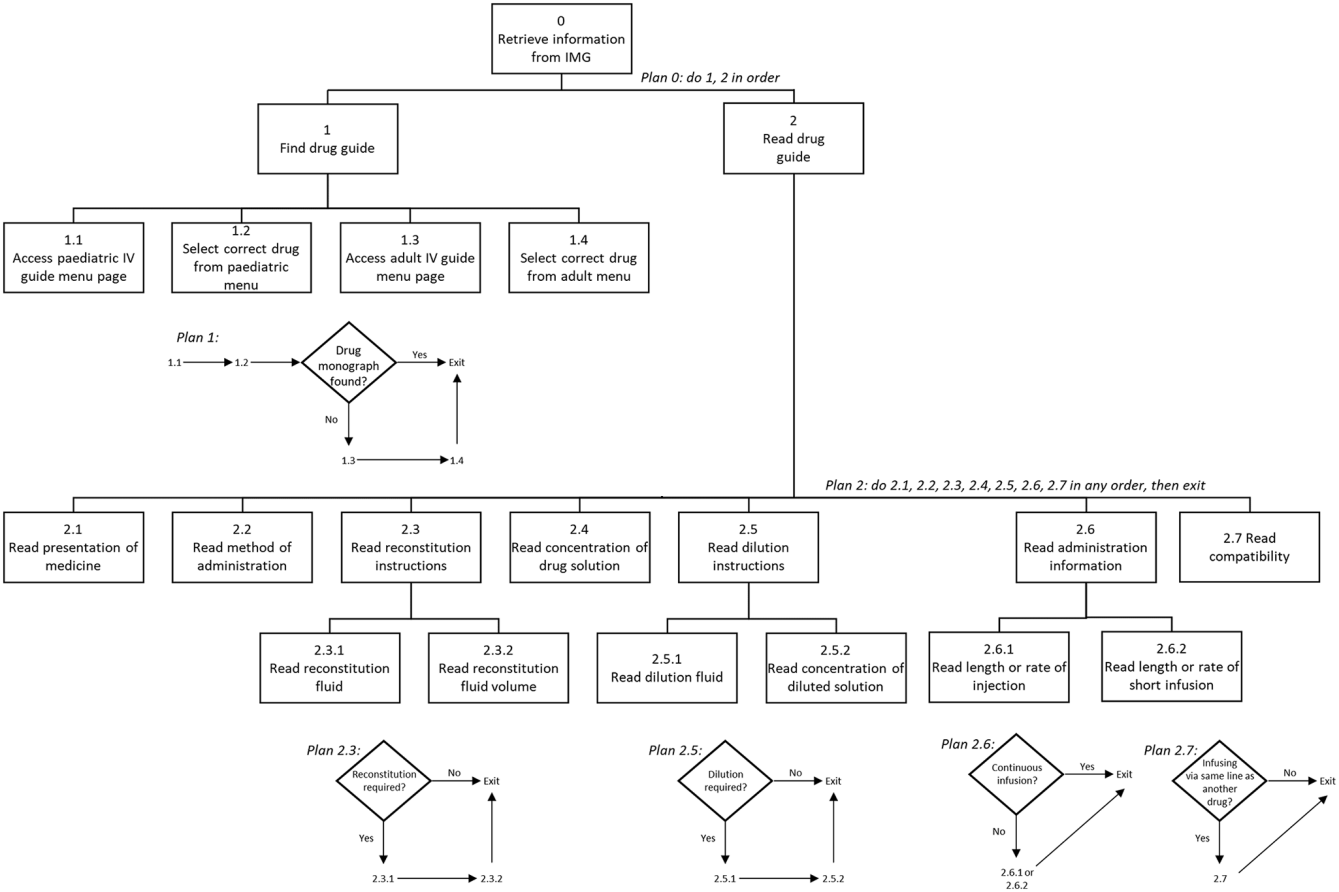
The hierarchical task analysis describing the process of retrieving all the information required for the preparation and administration of a pediatric intravenous medicine from the NHS IMG website. Abbreviations: IMG, Injectable Medicines Guide; IV, intravenous.

A new HTA was developed to describe the process of retrieving from the IMG all the information required for the preparation and administration of a pediatric intravenous medicine (Figure 1). The HTA was initially developed by a research pharmacist (MDJ) with experience of hospital pharmacy, the IMG, and injectable medicine safety research. It was subsequently assessed for face validity by 2 independent specialist pediatric pharmacists, an experienced pediatric nurse (CF), and a doctor (NA).

The IMG website is divided into pediatric and adult sections accessed via separate menu webpages. Guidance for different medicines is presented on individual webpages selected from either menu. The HTA (Figure 1), therefore, describes 2 major stages: finding the correct drug guide to use (steps 1.1-1.4) and subsequently reading that guide to extract the required information in any order (steps 2.1-2.7). At the time of data collection, the IMG did not contain pediatric guides for every medicine. For medicines without a specific pediatric guide, information regarding administration to children was presented within an “all age” guide, accessed via the adult menu page. This process is reflected in the “find drug guide” steps (1.1-1.4) of the HTA.

### Video Analysis

Video recordings of each of the 42 discrepancies that occurred during the “Check intravenous administration guidance” task of the original study^8^ were reanalyzed by a research pharmacist (MDJ). Observed deviations from expected practice at the level of an individual task (as described by the present study HTA; Figure 1) were defined as discrepancies and coded using a subset of error modes drawn from a generic human error taxonomy.^8^ When a participant sought but did not find relevant sections available within the IMG, the relevant process step was assigned the error mode “information not obtained” (error mode R1). When a participant found relevant sections within the IMG but subsequently extracted incorrect information from them, the relevant process step was assigned the error mode “wrong information obtained” (error mode R2). When a participant found relevant sections within the IMG but subsequently correctly extracted only some of the necessary information, the relevant process step was assigned the error mode “information retrieval incomplete” (error mode R3). When a participant did not attempt to use the IMG to obtain necessary information, the relevant process step was assigned the error mode “information not sought” (error mode R4). Where a single process step took longer than 1 minute to complete, it was assigned the error mode “operation took too long” (error mode A1). To capture the “root cause” of system vulnerabilities, an action that was performed correctly but that perpetuated a discrepancy that had already been made earlier was not classed as a discrepancy.

Each discrepancy was also classified according to its contribution to a subsequent MAE using the following categories: “no contribution” (the discrepancy did not contribute to a MAE), “minor contribution” (some contribution made to a MAE), and “major contribution” (the task discrepancy led directly to an MAE).

To determine the interobserver reliability, we agreed in advance that a minimum of 10% of videos should be analyzed in duplicate. Therefore, 5 videos (12%) were reanalyzed by an experienced pediatric nurse (CF), who considered observed discrepancies and assigned error modes and subsequent MAE contributions.

### Data Analysis

Counts of discrepancies were grouped by HTA task, error mode, and their contribution to MAEs. Discrepancy rates were calculated as percentages for each of the 3 medication error contribution categories (“no contribution,” “minor contribution,” and “major contribution”), with the number of observed discrepancies for each unique combination of HTA step and error mode as the denominator. The percentage of major contribution discrepancies contributing to a clinically significant and/or a large-magnitude MAE was also calculated.

## Results

The characteristics of the 60 participants in the 15 simulations are summarized in the original publication.^8^ Medicines were solely prepared and administered by the 30 nurses. Of these participants, 29 (97%) were female and 21 (70%) had fewer than 5 years’ experience of both general and pediatric practice.

For the 5 medication administrations that were reanalyzed by 2 researchers, there was perfect agreement for discrepancies, errors modes, and contribution to MAEs.

In total, 44 HTA step discrepancies in 33 doses were observed during use of the IMG. Of these discrepancies, 21 (48%) were linked to an MAE (identified in the original study^8^), with 16 (36%) making a major contribution to an MAE. Table 1 summarizes the discrepancy count for each unique HTA step and error mode combination (Figure 1). There were more discrepancies (31 in total, 70%) in the steps required to find the correct drug guide (steps 1.1-1.4) than there were in the steps required to read a drug guide (steps 2.1-2.7; 13 in total, 30%).

**Table 1.** Number, Frequency, and Relationship of Discrepancies and Error Modes to Resultant Administration Errors With Subanalysis of Discrepancies With a Major Contribution to Clinically Significant and Large-Magnitude Administration Errors.

HTA step at which discrepancy occurred	Error mode^a^	Description of discrepancy	Total discrepancies (n)	Relationship to resultant medication administration errors				
				No contribution n (%^b^)	Minor contribution n (%^b^)	Discrepancies that made a major contribution to a medication administration error		
						Major contribution, total, n (%^b^)	Discrepancies that resulted in clinically significant errors, n (%^b^)^c^	Discrepancies that resulted in large-magnitude errors, n (%^b^)^d^
**Overall**			**44**	**23 (52%)**	**5 (11%)**	**16 (36%)**	**6 (14%)**	**4 (9%)**
**Find drug guide**			**31**	**17 (55%)**	**4 (13%)**	**10 (32%)**	**4 (13%)**	**4 (13%)**
1.1: Access pediatric IV guide menu page	R1	Used adult guide when pediatric guide available	11	7 (64%)	—	4 (36%)	3 (27%)	3 (27%)
	R4	Did not check IMG for guidance	8	2 (25%)	—	6 (75%)	1 (13%)	1 (13%)
1.2: Select correct drug from pediatric menu	A1	>1 minute searching for correct guide	4	3 (75%)	1 (25%)	—	—	—
1.3: Access adult IV guide menu page	R4	Did not look for an all-age guide after finding no pediatric guide available	7	4 (57%)	3 (43%)	—	—	—
1.4: Select correct drug from adult menu	R1	Failed to open all-age guide	1	1 (100%)	—	—	—	—
**Read drug guide**			**13**	**6 (46%)**	**1 (8%)**	**6 (46%)**	**2 (15%)**	—
2.2: Read method of administration	R2	Misread method as inject aciclovir undiluted	1	—	—	1 (100%)	1 (100%)	—
	R3	Did not read that diluted phenytoin is “preferred”	1	—	—	1 (100%)	1 (100%)	—
	R4	Information not sought	1	—	—	1 (100%)	—	—
2.2.2: Read reconstitution fluid volume	R4	Information not sought	1	—	—	1 (100%)	—	—
2.4: Read concentration of drug solution	A1	>1 minute reading time	1	1 (100%)	—	—	—	—
	R4	Information not sought	1	—	—	1 (100%)	—	—
2.5.2: Read concentration of diluted solution	A1	>1 minute reading time	2	2 (100%)	—	—	—	—
	R3	Did not notice it was “maximum” dopamine concentration to dilute to	1	—	—	1 (100%)	—	—
2.6.1: Read length or rate of injection	R4	Information not sought	1	—	1 (100%)	—	—	—
2.6.2: Read length or rate of short infusion	A1	>1 minute reading time	1	1 (100%)	—	—	—	—
	R2	Read neonatal ceftriaxone infusion time instead of infant time	1	1 (100%)	—	—	—	—
2.7: Read compatibility	A1	>1 minute reading time	1	1 (100%)	—	—	—	—

Abbreviations: HTA, hierarchical task analysis; IMG, Injectable Medicines Guide; IV, intravenous.

a Error mode codes: A1, operation took too long; R1, information not obtained; R2, wrong information obtained; R3, information retrieval incomplete; R4, information not sought.

b The denominator for percentages is the total number of discrepancies in each row.

c Number and percentage of clinically significant administration errors (severity score > 3), with major contributory discrepancies made at each specific step, of a total of 22 clinically significant errors identified in the original study.^8^

d Number and percentage of large-magnitude administration errors (deviation from recommended dosing range or deviation from recommended dosing rate > 25%), with major contributory discrepancies made at each specific step, of a total of 27 large-magnitude errors identified in the original study.^8^

Bold faces represent totals and sub-totals of the figures which follow in subsequent rows.

Accessing the pediatric intravenous guide (step 1.1) was the step with the most discrepancies (n = 19) and the step most likely to contribute to MAEs, with 10 major contributions, including 4 that were clinically significant. These included 11 discrepancies resulting from participants using the adult IMG when a pediatric version was available. These 11 discrepancies were distributed between only 5 teams (2 teams with 1 discrepancy each, and 3 teams with 2, 3, and 4 discrepancies, respectively). Teams with multiple discrepancies of this type continued to use the adult IMG for medicines subsequent to the first discrepancy because use of the “back” button of their web browser meant the next drug guide selection was also made from the adult menu. Across all steps, the most common error modes were “information not sought” (R4, 19 discrepancies, 43%), “information not obtained” (R1, 12 discrepancies, 27%), and “operation took too long” (A1, 9 discrepancies, 20%).

Overall, there were 6 discrepancies that made a major contribution to a clinically significant MAE and 4 that made a major contribution to a large-magnitude MAE (Table 1). These are described in more detail in Table 2. This is equivalent to 27% and 15% of all clinically significant and large-magnitude medication errors observed in the original study, respectively. All discrepancies making a major contribution to a large-magnitude administration error arose at the step of accessing the pediatric intravenous guide (step 1.1). A further 2 clinically significant administration errors arose from misreading the method of administration of a medication (step 2.2).

**Table 2.** Description of the Discrepancies That Made a Major Contribution to a Clinically Significant or Large-Magnitude Medication Administration Error.

Medicine	HTA step at which discrepancy occurred^a^	Error mode^b^	Mean severity score^c^	Deviation from recommended dosing range^c^	Deviation from recommended administration rate^c^	Description of related medication administration error	Description of contributory IMG-related discrepancy
Calcium chloride	1.1	R1	8.6	789%	67%	8.8 mmol given undiluted over 3 minutes rather than 0.99 mmol given diluted over 5-10 minutes	Used the adult IMG, which suggested administration of undiluted injection over 5 minutes. Pediatric IMG would have suggested diluting the injection and giving over 5-10 minutes. (Incorrect dose was based on mis-reading of the BNF)
Phenytoin	1.1	R1	7.2	—	300%	Correct dose given undiluted at 4 mg/kg/min rather than diluted at 1 mg/kg/min	Used the adult IMG, which did not state that dilution was preferred and gave a maximum infusion rate of 50 mg/min. Pediatric IMG would have stated that dilution was preferred and gave a maximum infusion rate of 1 mg/kg/min
Phenytoin	1.1	R1	6.9	—	−99%	Correct dose given too slowly (0.0078 mg/kg/min rather than 1 mg/kg/min)	Used the adult IMG, which suggested administration rate of 25 mg/min in “some patients.” Decided to give at 25 mg/min but made a calculation error when setting rate on pump. Pediatric IMG would have suggested a maximum infusion rate of 1 mg/kg/min
Lorazepam	1.1	R4	5.4	300%	High	3.2 mg given over a few seconds rather than 0.8 mg over 3-5 minutes	Did not use the IMG, which would have stated that dose should be given over 3-5 minutes
Aciclovir	2.2	R2	5.7	—	High	Correct dose given undiluted over a few seconds, rather than diluted over 1 hour	Misinterpreted the “Method of administration” section as saying that aciclovir should be given undiluted by short injection, possibly as a result of misreading “Do not administer by IV injection.” (Was also using the adult IMG, but pediatric IMG had the same wording, so this did not contribute to the error)
Phenytoin	2.2	R3	4.4	—	—	Correct dose and rate given undiluted rather than diluted	Did not notice that the “Method of administration” section in the pediatric IMG stated that diluted infusion is preferred

Abbreviations: BNF, British National Formulary; HTA, hierarchical task analysis; IMG, Injectable Medicines Guide; IV, intravenous.

a 1.1 = “Access pediatric IV guide menu page”; 2.2 = “Read method of administration.”

b Error mode codes: R1 = information not obtained; R2 = wrong information obtained; R3 = information retrieval incomplete; R4 = information not sought.

c Derived from the original study.^9^

## Discussion

This study has identified the specific steps in the process of using the IMG that contributed to MAEs during simulated pediatric resuscitations. Process discrepancies were most frequent in the steps required to find the correct drug guide but were also seen during the extraction of correct and complete information from individual guides. Many of these discrepancies were important because more than one-third made a major contribution to a subsequent MAE and the process of retrieving information from the IMG made a major contribution to more than a quarter of clinically significant errors observed in the simulation study. There were 3 process discrepancies that made a major contribution to clinically significant and/or large-magnitude MAEs: relying on memory rather than checking the IMG for information, using an adult guide instead of a pediatric guide, and misinterpreting method of administration information (Table 2). The design of the IMG also meant that a “use of an adult guide” discrepancy was likely to lead to subsequent similar administration errors. In addition, 9 discrepancies (20%) were coded with error mode A1: “operation took too long.” This suggests that the IMG might be less suitable for use in time-critical scenarios, although this did not significantly contribute to MAEs in this study. Since the present study was completed, the design of the IMG has been revised to reduce the likelihood of similar discrepancies occurring.

A previous study applied user testing to the adult IMG and found that nurses had similar difficulties to those reported in the present study in extracting correct and complete information.^12^ A subsequent ward-based simulation found that a new version of the IMG, revised via a user testing process, resulted in approximately 2.5 times more medication administrations being free of guideline-related errors.^16^ Nurses were also able to prepare intravenous medicines more quickly when using the user-tested guidelines. However, these studies did not consider use of the pediatric IMG nor the process of finding the correct drug guide, which most frequently contributed to MAEs in the present study. Nonetheless, the reduction in MAEs seen after application of a systematic approach to obtain and respond to feedback from users suggests that usability testing^17^ of the IMG website might be successful in preventing some of the errors observed. In addition, the results of this study provide another example of how difficulty in finding relevant, unambiguous information in any guideline can contribute to medication errors^5-7^ and to patient safety incidents more generally.^18,19^ By using HRA, the present study provides more information on which of the steps in using a particular guideline are associated with MAEs. More broadly, these findings fit with the wider literature on the use of electronic systems in health care, which shows that usability problems can contribute to medication errors and patient harm.^20-24^

Among the strengths of the present study are the use of HRA to link specific discrepancies to subsequent MAEs and the validation of the video analysis by a second researcher. However, several limitations are shared with the original study, including the use of a simulated environment at a single site with unblinded participants.^8^ Participation in a simulation may have changed nurses’ behavior compared with actual practice, thus reducing external validity. However, this effect may also exist in observational studies of clinical practice because of the potential effects of an observer and participation in a research study.^25^ In addition, although each simulation team included 1 senior nurse (defined by an appropriate UK salary band), the professional experience of the participants was less than might be expected, with 70% having fewer than 5 years’ experience. There are also more specific limitations. The use of the IMG was only observed during pediatric resuscitation scenarios, so the findings may have limited generalizability to less urgent situations involving medicines that are more commonly used or less complex to prepare. In particular, discrepancies involving selection of a drug guide for the wrong patient age group are less likely to be seen when the IMG is used by nurses caring for adults.

## Conclusion and Relevance

Process discrepancies in the use of an online injectable medicines guideline were often associated with subsequent MAEs, including those with potentially significant consequences. The most error-prone steps were those related to finding the guideline for the correct age group, but discrepancies were also seen during the subsequent extraction of correct and complete information from the guidelines.

These findings suggest that work to prevent MAEs related to the IMG should focus on encouraging nurses to use the IMG to find guidelines on intravenous medicines, ensuring that adult guides are not used when administering medicines to children (and that this discrepancy is not perpetuated by default), and improving the clarity of the method of administration guidance. Refinements to the design of the IMG (some of which have already been implemented) are likely to bring about robust improvements, but raising staff awareness of these common discrepancies may also help while design changes are implemented. In the longer term, the integration of the IMG into electronic prescribing and medicines administration systems could remove the need for staff to search for the correct information, thus removing the most problematic process steps observed in this study.

More widely, these findings and those of other studies suggest that the authors and designers of guidelines and other electronic tools should consider the usability of their products (including both navigation and interpretation) as well as their accuracy and comprehensiveness.^12,16^ Tools such as user testing may be helpful in achieving this aim. Quantitative HRA has been shown to be a valuable method by which to measure the vulnerability of clinical guidelines to misuse. Future research should examine the use of guidelines for other clinical applications and in other clinical areas to determine whether certain types of discrepancy are common between different guideline types and intended audiences. It should also seek to clarify the contribution that tools such as user testing can make to the prevention of medication errors related to guidelines and other electronic systems.
